# Supplementary material for: Lipogenic enzyme FASN promotes mutant p53 accumulation and gain-of-function through palmitoylation
Source: Nat Commun. 2025 Feb 19;16:1762. doi: 10.1038/s41467-025-57099-9 (PMC11839913; doi:10.1038/s41467-025-57099-9)
Supplement: Supplementary file 1 — Supplementary Information [file 41467_2025_57099_MOESM1_ESM.pdf]

Supplementary Materials:

Supplementary Table 1

TVB-3166 treatment does not affect the tumor spectrum of *p53* $^{R172H/R172H}$ mice

Tumor type	Control (n=19 mice)	TVB-3166 (n=18 mice)
Thymic lymphoma	13	11
Splenic lymphoma	7	8
Liposarcoma	1	1
Osteosarcoma	1	0
Number of tumors	22	20

Supplementary Table 2. Sequences of siRNAs and sgRNAs

Target gene		Sequence
FASN siRNA	# 1	5’- CGAGAGCACCTTTGATGAC -3’
	# 2	5’- CATGGAGCGTATCTGTGAG -3’
Mouse *FASN* siRNA	# 1	5’- TTGCCAATCTCTAAGAAGC -3’
	# 2	5’- TTATGAAGAAGCATAAGGC -3’
p53 siRNA	# 1	5’- GACTCCAGTGGTAATCTACT -3’
	# 2	5’- GTCCAGATGAAGCTCCCAGAA -3’
p53 sgRNA	# 1	5’- CCATTGTTCAATATCGTCCG -3’
	# 2	5’- GGGCAGCTACGGTTTCCGTC -3’
FASN sgRNA	# 1	5’- TGCCCTTCCG TGGCTACGCT -3’
	# 2	5’- CGATGTCGTTCAGCATGCTC -3’

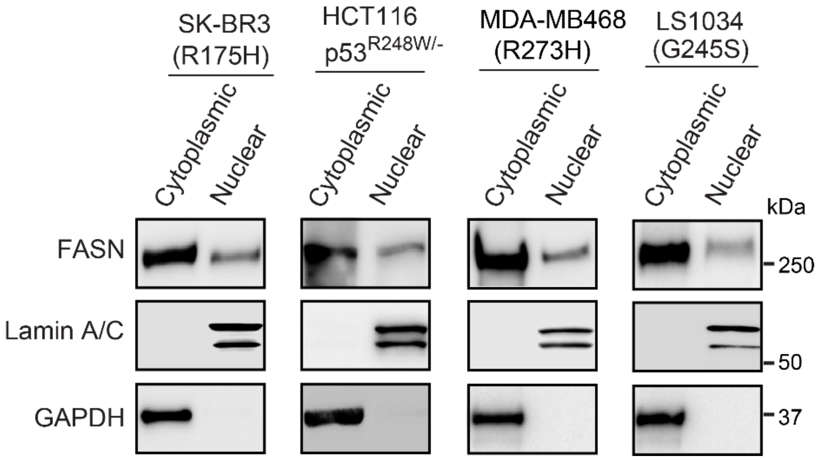

Supplementary Fig. 1. FASN is detected in both the cytoplasmic and nuclear fractions of different cancer cell lines. The cytoplasmic and nuclear fractions of SK-BR3, *p53*$^{R248W/-}$ HCT116, MDA-MB468, and LS1034 cells were isolated and subjected to Western blot assays. Lamin A/C and GAPDH were used as markers for the nuclear and cytoplasmic fractions, respectively. Data represent three repeats with similar results. Source data are provided as a Source Data file.

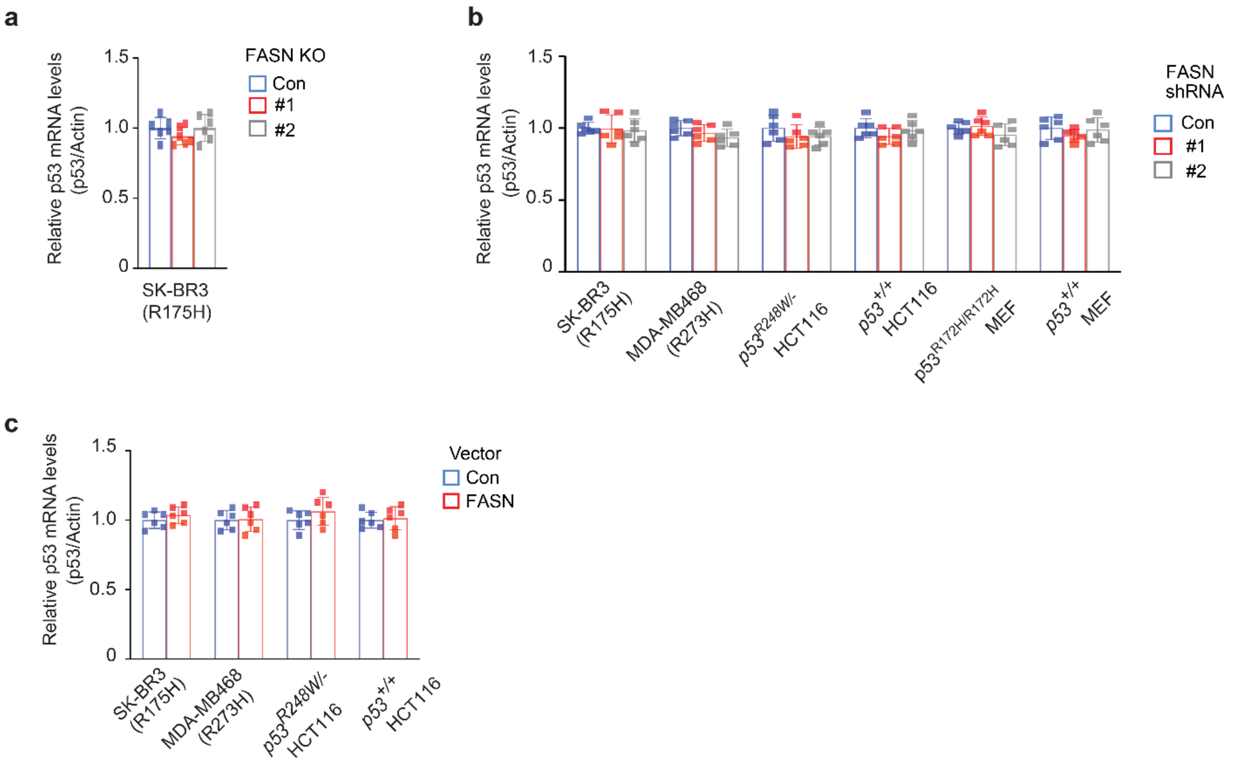

Supplementary Fig. 2. FASN expression does not affect the mRNA levels of mutp53 or wtp53 in different cell lines. **a**, FASN KO did not affect mutp53 mRNA levels in SK-BR3 cells. **b**, FASN knockdown by shRNA vectors did not affect the mRNA levels of mutp53 or wtp53 in different cell lines. **c**, Ectopic expression of FASN-Flag did not affect the mRNA levels of mutp53 or wtp53 in cells. In **a**-**c**, the mRNA levels of mutp53 or wtp53 in cells were measured by TaqMan real-time PCR assays and normalized with Actin. The FASN expression did not show significant effect on mutp53 or wtp53 mRNA levels in these cell lines. Data represent mean ± SD (n = 6 independent experiments). One-way ANOVA followed by Dunnett's test (**a** and **b**) or two-tailed unpaired Student's *t*-test (**c**). Source data are provided as a Source Data file.

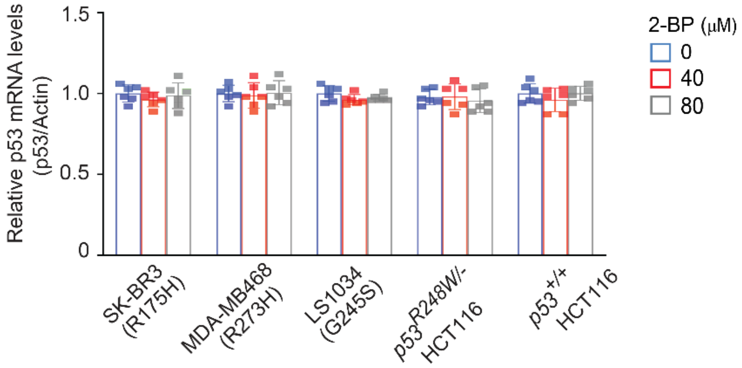

Supplementary Fig. 3. 2-BP treatment does not affect the mRNA levels of mutp53 or wtp53 in different cell lines. The mRNA levels of mutp53 or wtp53 in cells treated with or without 2-BP (40 or 80 µM) for 24 h were measured by TaqMan real-time PCR assays and normalized with Actin. 2-BP treatment did not significantly affect the mRNA levels of mutp53 or wtp53 in different cell lines. Data represent mean ± SD (n = 6 independent experiments). One-way ANOVA followed by Dunnett's test. Source data are provided as a Source Data file.

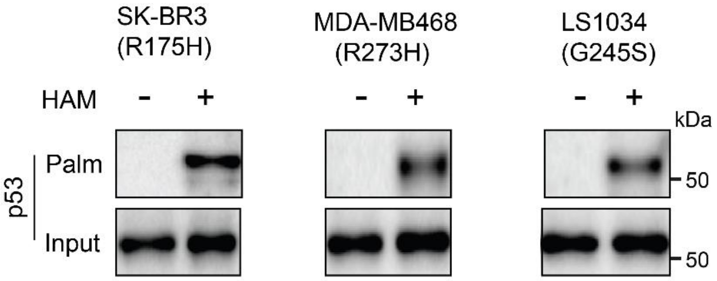

Supplementary Fig. 4. ABE analysis of the palmitoylation of endogenous mutp53 in different cell lines. Different cell lines carrying different endogenous mutp53 were used for ABE analysis of the mutp53 palmitoylation. To enable the standardized comparison of the p53 palmitoylation levels across various samples, the input mutp53 levels were normalized by adding different amounts of protein samples for Western blot analysis. Palm p53: palmitoylated p53. HAM: hydroxylamine. Data represent three repeats with similar results. Source data are provided as a Source Data file.

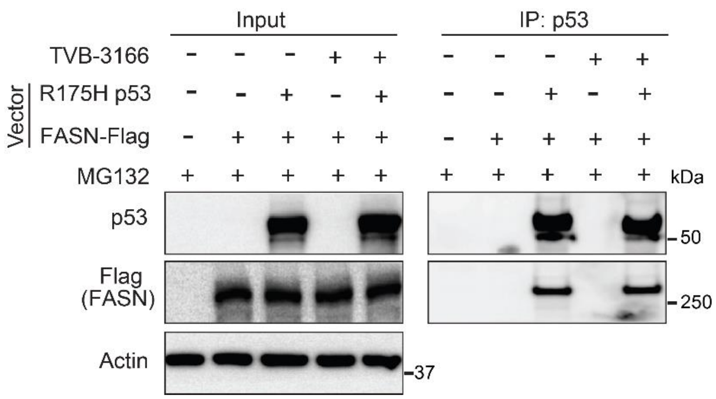

Supplementary Fig. 5. TVB-3166 treatment does not affect the mutp53-FASN interaction in cells. *p53*$^{-/-}$ HCT116 cells with or without ectopic expression of R175H mutp53 and/or FASN-Flag were subjected to TVB-3166 treatment (40 μM for 24 h) before the co-IP analysis. Cells were treated with proteasomal inhibitor MG132 (10 μM for 12 h) to inhibit ubiquitination and degradation of mutp53 protein to avoid that different amounts of p53 protein in cells affect its interaction with FASN. Data represent three repeats with similar results. Source data are provided as a Source Data file.

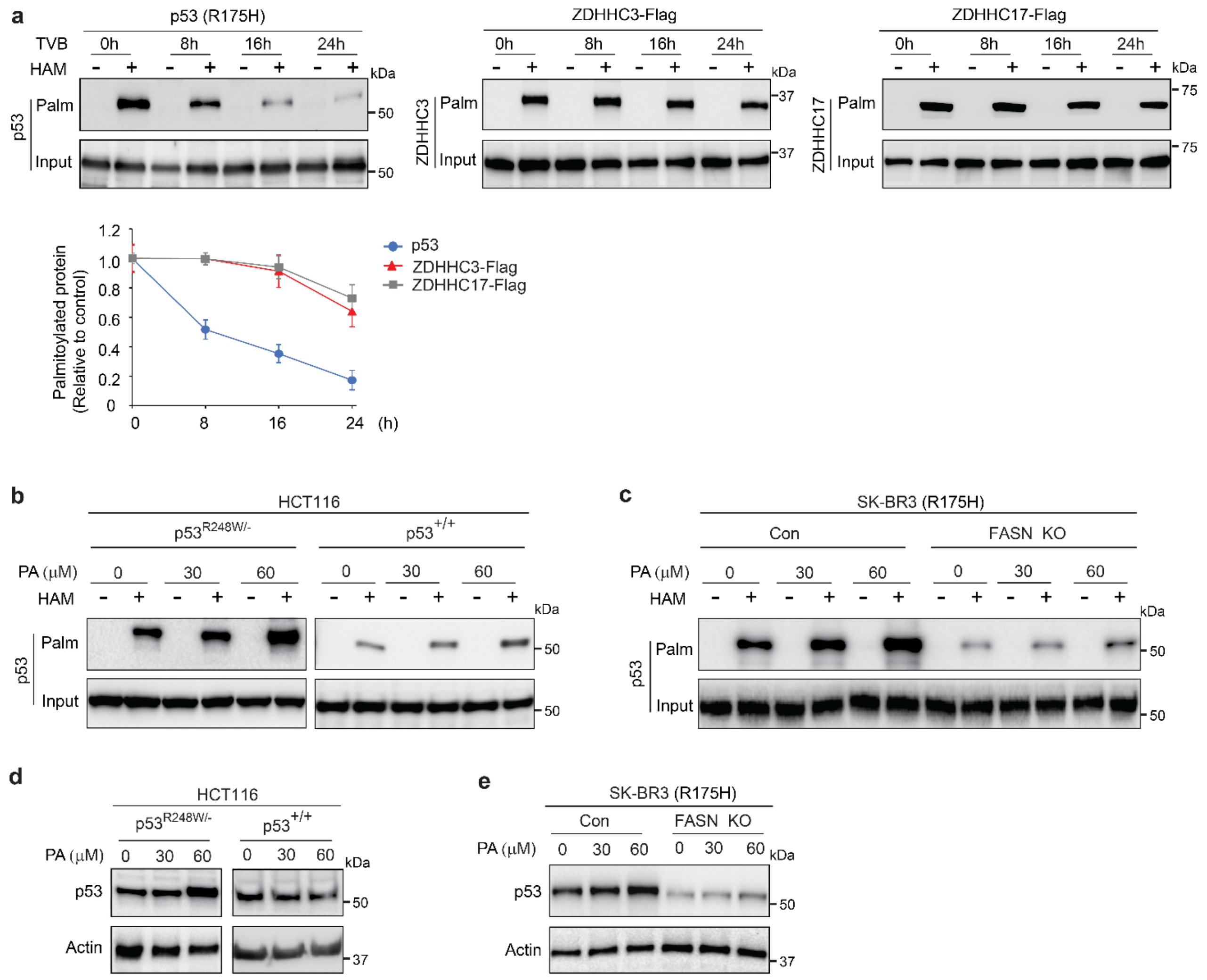

Supplementary Fig. 6. The effects of TVB-3166 and exogenous palmitate on mutp53 palmitoylation and protein levels. **a,** Inhibition of FASN by TVB-3166 displayed a faster inhibitory effect on the palmitoylation of mutp53 than that of ZDHHC3 or ZDHHC17 in cells. *p53*$^{-/-}$ HCT116 cells with ectopic expression of R175H mutp53, ZDHHC3, or ZDHHC17 were treated with the TVB-3166 (40 μM) for the indicated hours (h) before ABE analysis of the palmitoylation of mutp53, ZDHHC3, and ZDHHC17. Lower panel: The relative levels of palmitoylated mutp53, ZDHHC3, and ZDHHC17 in cells treated with TVB-3166 for the indicated hours compared with control cells without TVB-3166 treatment (0 h). Data represent mean ± SD (n = 3 independent experiments). **b, c,** The effect of exogenous palmitate on mutp53 palmitoylation in HCT116 (**b**) and SK-BR3 cells (**c**). *p53*$^{+/+}$ and *p53*$^{R248W/-}$ HCT116 cells (**b**) and SK-BR3 cells with or without FASN KO (**c**) were treated with palmitate at the indicated concentrations for 24 h before ABE analysis of mutp53 palmitoylation. To enable the standardized comparison of p53 palmitoylation levels across various samples, the input p53 levels were normalized by adding different amounts of protein samples for Western blot analysis. **d, e,** The effect of exogenous palmitate on mutp53 protein levels in HCT116 (**d**) and SK-BR3 cells (**e**). *p53*$^{+/+}$ and *p53*$^{R248W/-}$ HCT116 cells (**d**) and SK-BR3 cells with or without FASN KO (**e**) were treated with palmitate at the indicated concentrations for 24 h before Western blot analysis. PA: palmitate. Data represent three repeats with similar results. Source data are provided as a Source Data file.

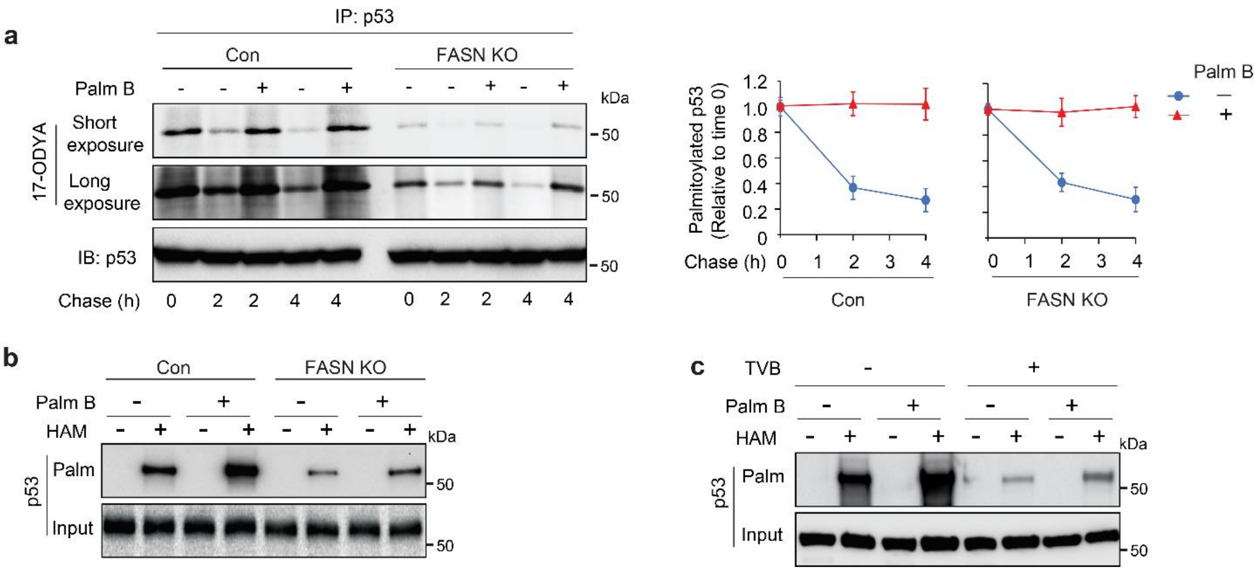

Supplementary Fig. 7. FASN does not affect the mutp53 depalmitoylation. a, Pulse-chase analysis of mutp53 depalmitoylation by click chemistry in cells with or without FASN KO. *p53*$^{-/-}$ HCT116 cells with ectopic expression of R175H mutp53 were labeled with 17-ODYA (30 µM) for 2 h, and then chased for the indicated times with either DMSO (-) or the APT1 inhibitor Palmostatin B (10 µM) to block depalmitoylation. The whole cell lysates were then subjected to click chemistry. Right panels: quantification of the palmitoylated mutp53. Data represent mean ± SD (n = 3 independent experiments). **b**, Palmostatin B increased the palmitoylation of mutp53 in cells with or without FASN KO as determined by ABE assays. **c,** Palmostatin B increased the palmitoylation of mutp53 in cells treated with or without TVB-3166 treatment (40 µM for 24 h) as determined by ABE assays. In **a-c**, to enable the standardized comparison of the p53 palmitoylation levels across various samples, the input p53 levels were normalized by adding different amounts of protein samples for Western blot analysis. Palm B: Palmostatin B. TVB: TVB-3166. Data represent three repeats with similar results. Source data are provided as a Source Data file.

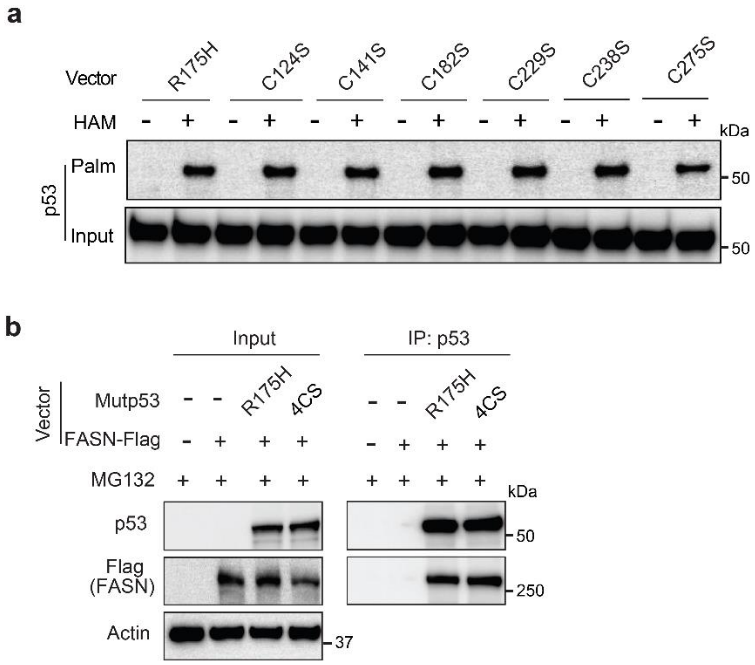

Supplementary Fig. 8. Identification of mutp53 palmitoylation sites. **a**, Identification of mutp53 palmitoylation sites by ABE assays in *p53*$^{-/-}$ HCT116 cells expressing R175H mutp53 or its different CS (cysteine to serine) mutants. To enable the standardized comparison of the p53 palmitoylation levels across various samples, the input mutp53 levels were normalized by adding different amounts of protein samples for Western blot analysis. Palm p53: palmitoylated p53. HAM: hydroxylamine. **b**, 4CS mutations did not affect the interaction between mutp53 and FASN. Co-IP assays were performed in *p53*$^{-/-}$ HCT116 cells co-expressing FASN-Flag with either R175H mutp53 or 4CS mutp53. -: control vectors. Cells were treated with the proteasomal inhibitor MG132 (10 μM for 12 h) to inhibit ubiquitination and degradation of mutp53 protein to avoid that different amounts of p53 protein in cells affect its interaction with FASN. Data represent three repeats with similar results. Source data are provided as a Source Data file.

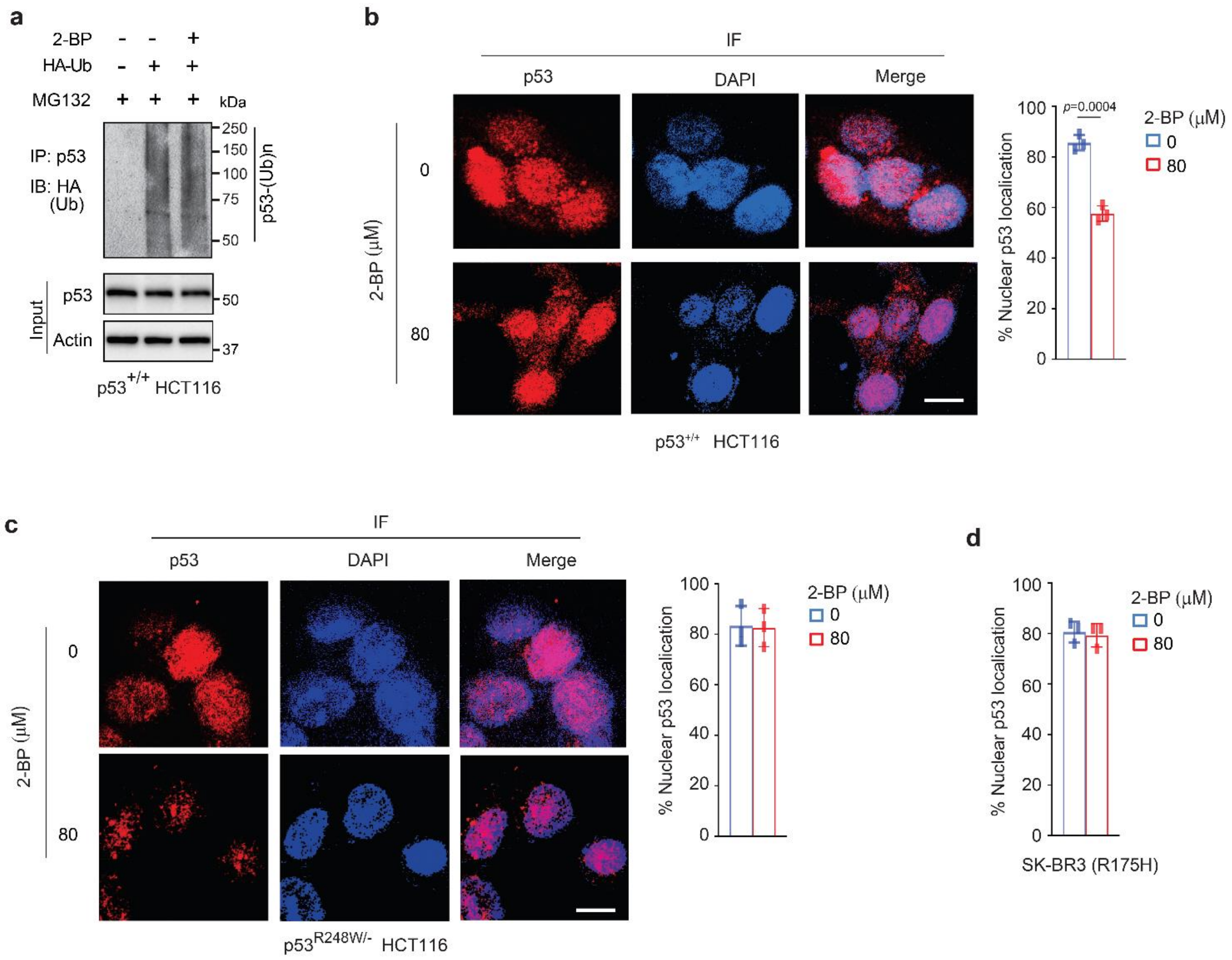

Supplementary Fig. 9. The effects of 2-BP treatment on the ubiquitination and nuclear localization of wtp53 in cells. **a,** The palmitoylation inhibitor 2-BP treatment did not affect the ubiquitination of wtp53 analyzed by ubiquitination assays in *p53*$^{+/+}$ HCT116 cells. **b,** 2-BP treatment reduced the nuclear localization of wtp53 in *p53*$^{+/+}$ HCT116 cells determined by IF staining. **c,** 2-BP treatment did not affect the nuclear localization of mutp53 in *p53*$^{R248W/-}$ HCT116 cells determined by IF staining. In **b**, **c**, left panels: representative IF images. Scale bar: 10 µm. Right panels: quantification of cells with the nuclear localization of wtp53 or mutp53 in cells. It is worth noting that 2-BP did not clearly affect wtp53 protein levels in *p53*$^{+/+}$ HCT116 cells, but clearly reduced mutp53 protein levels in *p53*$^{R248W/-}$ HCT116 cells as determined by IF staining. **d**, 2-BP treatment did not clearly affect the nuclear localization of mutp53 in SK-BR3 cells determined by IF staining. In **a-d,** cells were treated with 2-BP (80 µM) for 24 h before assays. In **b-d**, 200 cells were used for quantification for each independent experiment. Data represent mean ± SD (n = 3 independent experiments). Two-tailed unpaired Student's *t*-test. Data represent three repeats with similar results. Source data are provided as a Source Data file.

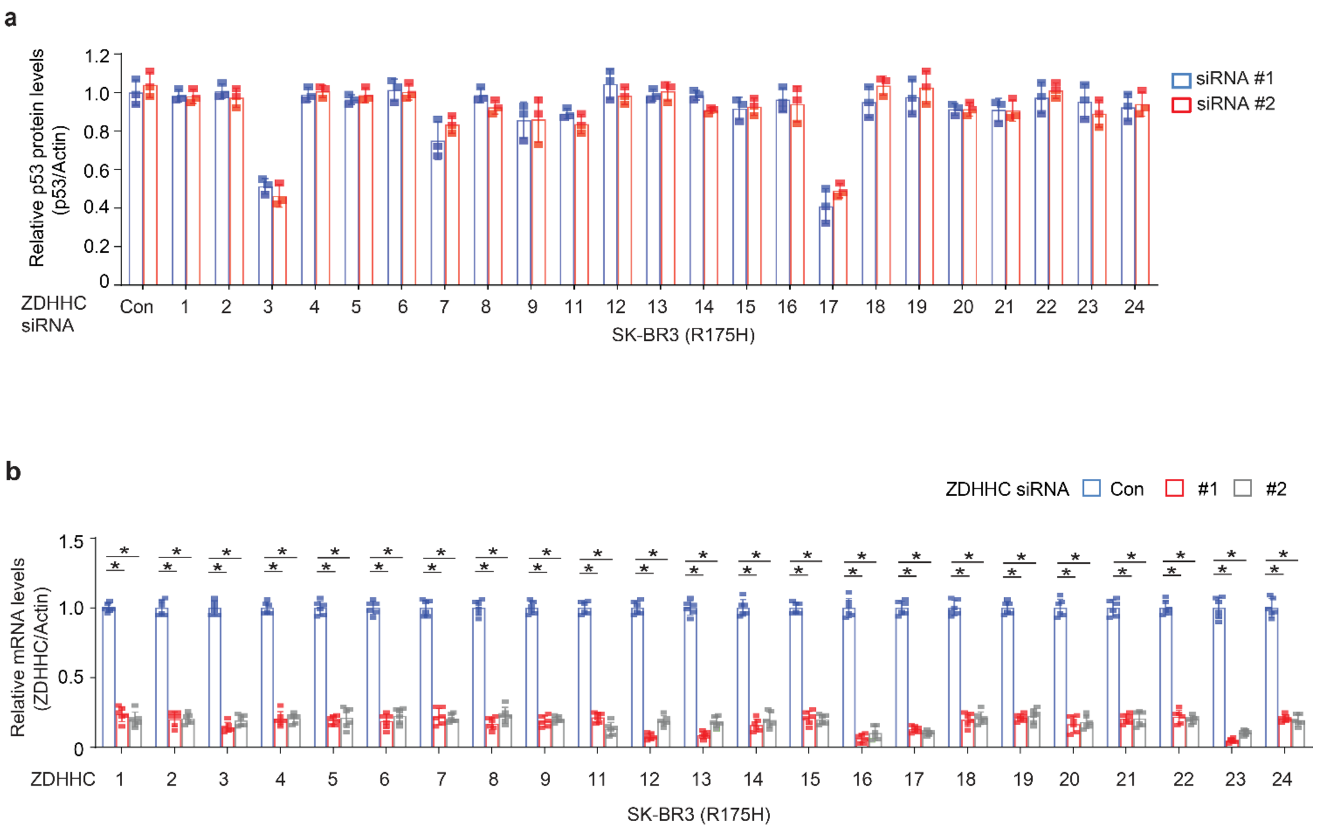

Supplementary Fig. 10. Identification of ZDHHC3 and ZDHHC17 as the major palmitoyltransferases of mutp53 by siRNA screening. a, b, Knockdown of ZDHHC3 or ZDHHC17 by siRNAs clearly reduced mutp53 protein levels in cells. SK-BR3 cells were transfected with 2 different siRNAs against each of 23 ZDHHC palmitoyltransferases. Mutp53 protein levels were analyzed by Western blot assays (**a**), and knockdown of ZDHHCs by siRNAs were analyzed by TaqMan real-time PCR assays (**b**). Data represent mean ± SD (n = 3 independent experiments in **a**; n=6 independent experiments in **b**). One-way ANOVA followed by Dunnett's test. *: p<0.0001. Source data are provided as a Source Data file.

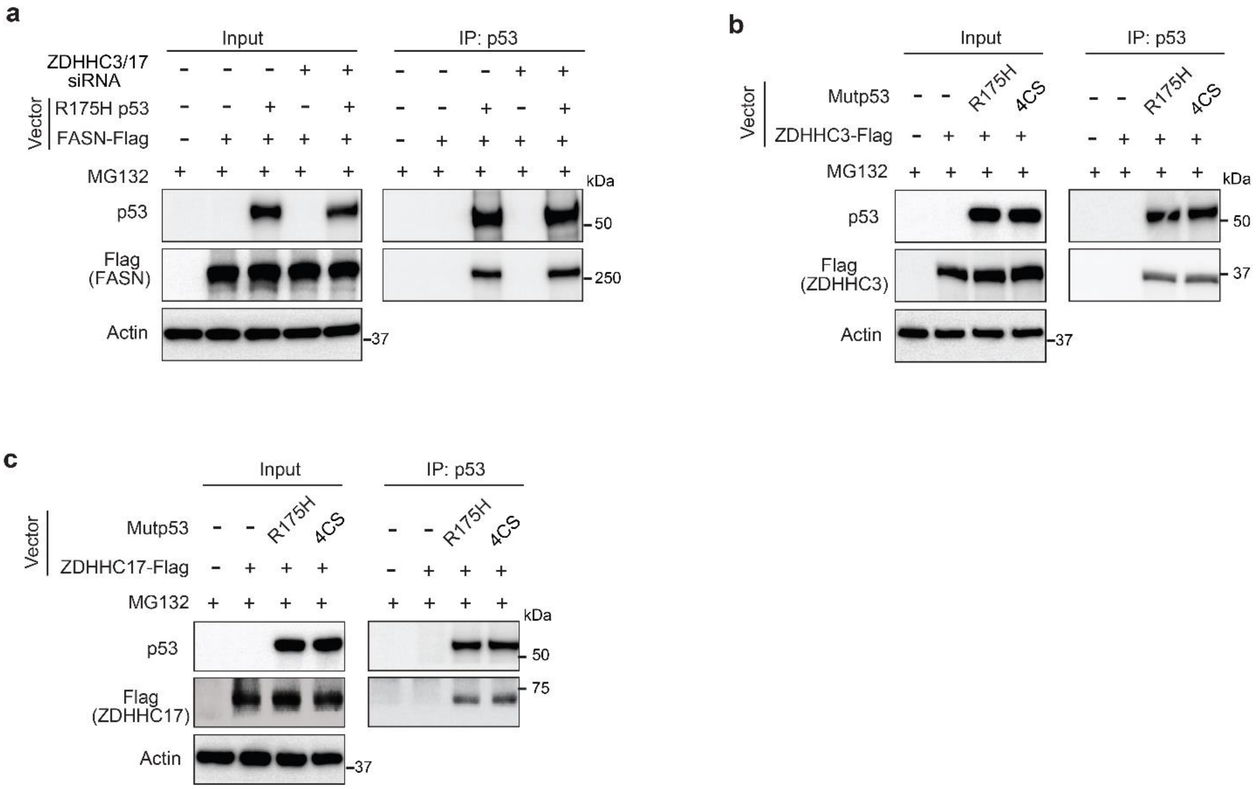

Supplementary Fig. 11. The impact of ZDHHC3/17 on mutp53-FASN interaction and the impact of 4CS mutations on mutp53-ZDHHC3/17 interaction. **a,** Knockdown of ZDHHC3 and ZDHHC17 together by siRNAs did not affect the interaction between mutp53 and FASN in *p53*$^{-/-}$ HCT116 cells. Two different siRNAs against ZDHHC3 and ZDHHC17, respectively, were used and similar results were observed. The efficiency of knockdown was confirmed in Fig. 4b. **b, c**, ZDHHC3 (**b**) and ZDHHC17 (**c**) interacted with both R175H mutp53 and 4CS mutp53 in cells. *p53*$^{-/-}$ HCT116 cells co-expressing ZDHHC3-Flag or ZDHHC17-Flag together with R175H mutp53 or 4CS mutp53 were employed for co-IP assays. -: control vectors. In **a-c**, cells were treated with the proteasomal inhibitor MG132 (10 μM for 12 h) to inhibit the ubiquitination and degradation of mutp53 protein to avoid that different amounts of p53 protein in cells affect its interactions with FASN and ZDHHC3/17. Data represent three repeats with similar results. Source data are provided as a Source Data file.

a

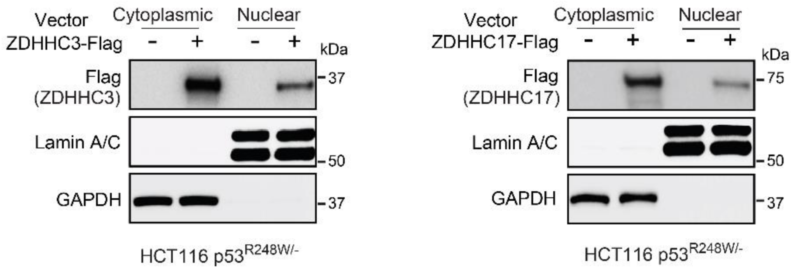

b

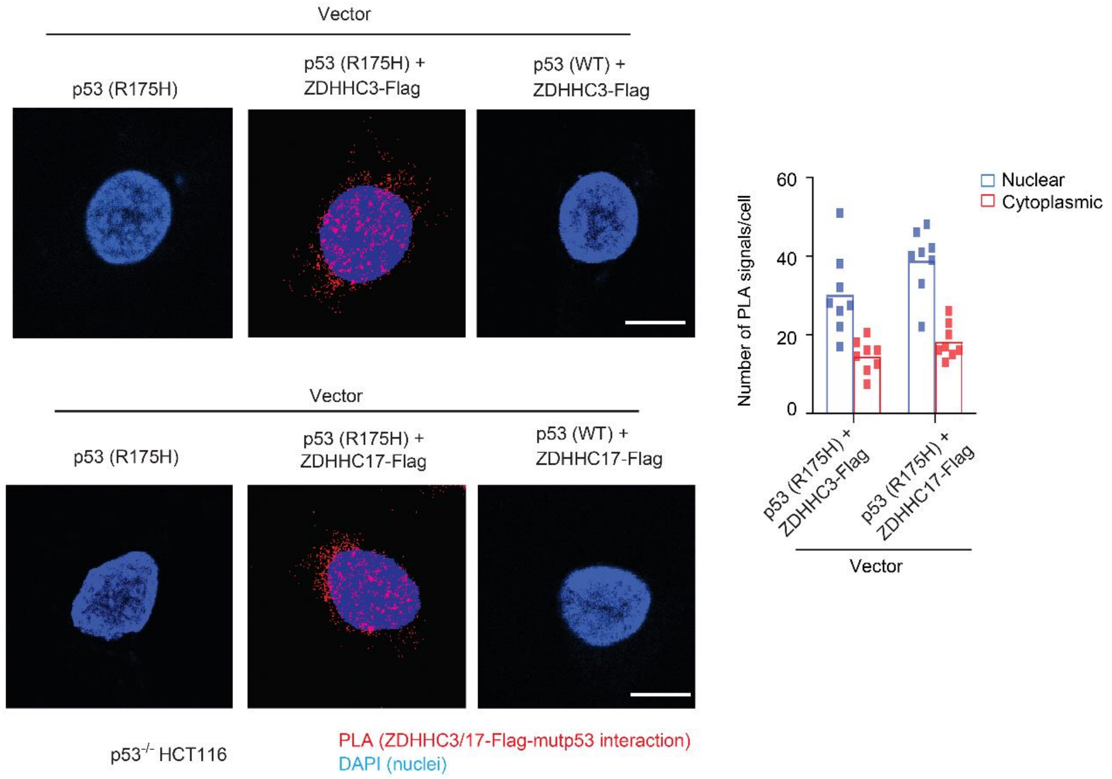

Supplementary Fig. 12. The interaction between ZDHHC3/17 and mutp53 in both cytoplasmic and nuclear compartments with more interactions detected in the nuclear compartment. a, The cytoplasmic and nuclear fractions of *p53*$^{R248W/-}$ HCT116 cells with or without ectopic expression of ZDHHC3 or ZDHHC17 were isolated and subjected to Western blot assays. Lamin A/C and GAPDH were used as markers for the nuclear and cytoplasmic fractions, respectively. **b**, The *in situ* interaction between the ectopic R175H mutp53 and ZDHHC3/17 proteins in *p53*$^{-/-}$ HCT116 cells analyzed using the proximity ligation assay (PLA). Scale bar: 10 μm. Representative images of PLA (Left panels) and quantification of PLA (right panel; number of PLA signals/cell) are shown. DAPI was used for the nuclear staining. Totally 200 cells were counted in 8 different areas from 3 independent experiments. Data represent three repeats with similar results. Source data are provided as a Source Data file.

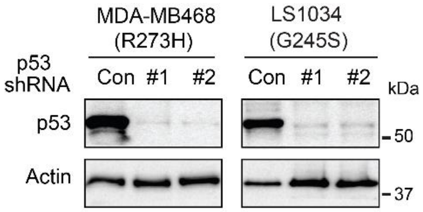

Supplementary Fig. 13. Mutp53 knockdown in cells. The knockdown of endogenous mutp53 by shRNA vectors in MDA-MB468 and LS1034 cells was confirmed at the protein levels by Western blot assays. Data represent three repeats with similar results. Source data are provided as a Source Data file.

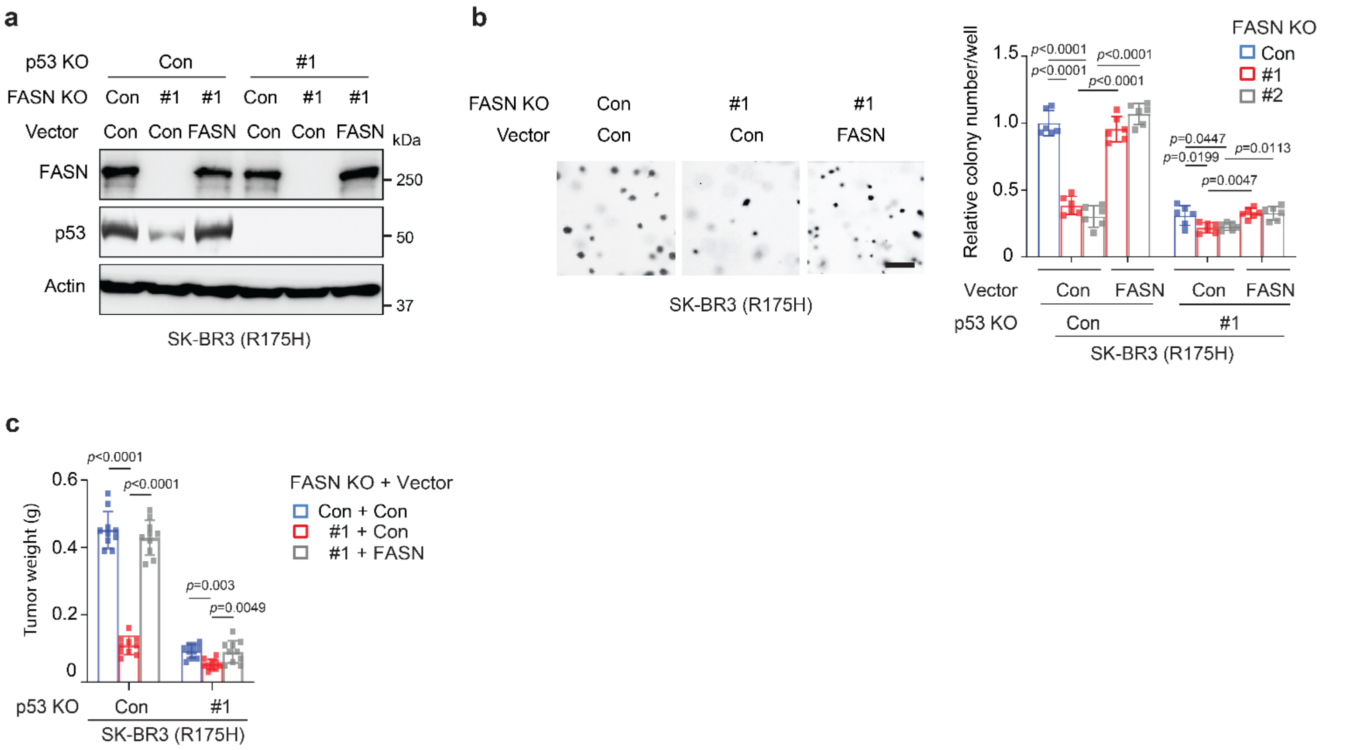

Supplementary Fig. 14. Ectopic expression of FASN rescues the impaired anchorage-independent growth (AIG) and tumorigenesis of cells resulting from FASN KO. a, Ectopic expression of FASN in cells with KO of endogenous FASN rescued the downregulation of mutp53 protein levels in SK-BR3 cells. SK-BR3 cells with FASN KO were stably transduced with FASN expression vectors to increase the level of FASN protein to a comparable level as endogenous FASN in control SK-BR3 cells transduced with control vectors. Data represent three repeats with similar results. Cells were then used for assays in **b** and **c**. **b,** Ectopic FASN expression rescued the impaired AIG in soft agar of SK-BR3 cells resulting from FASN KO. Left: Representative images of AIG of cells. Scale bar: 200 µm. **c,** Ectopic FASN expression rescued the impaired growth of SK-BR3 orthotopic tumors in nude mice resulting from FASN KO. Data represent mean ± SD (n = 6 independent experiments in **b** and n=10 mice/group in **c**). One-way ANOVA followed by Tukey's test. Source data are provided as a Source Data file.

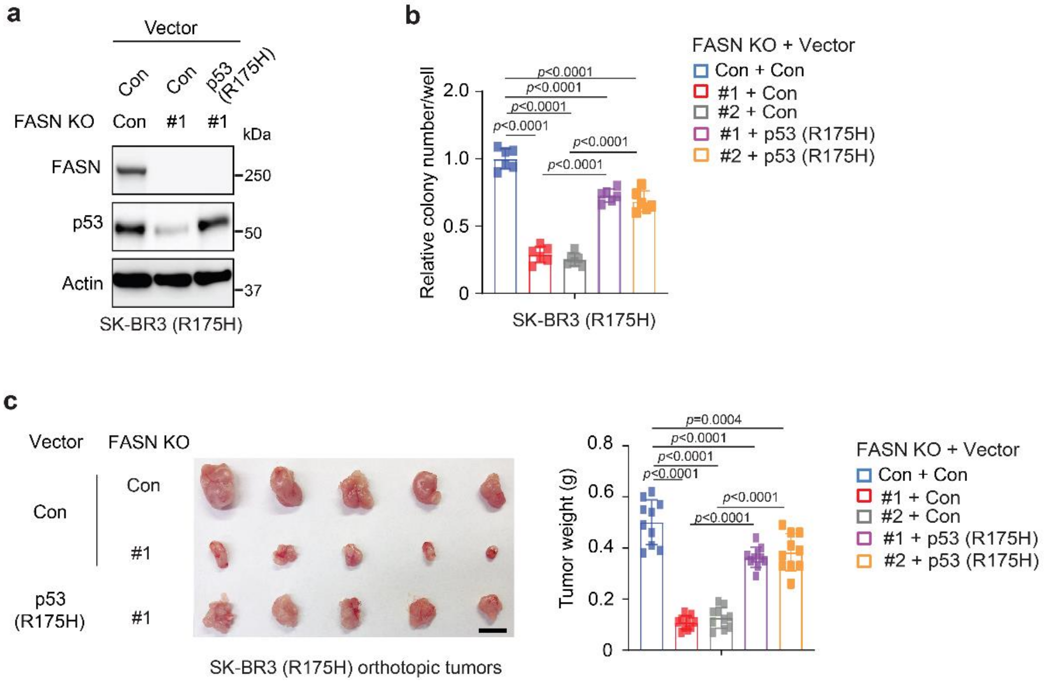

Supplementary Fig. 15. The ectopic expression of mutp53 partially rescues the impaired anchorage-independent growth (AIG) and tumorigenesis resulting from FASN KO. a, Ectopic expression of R175H mutp53 in SK-BR3 cells with KO of endogenous FASN. SK-BR3 cells with FASN KO were stably transduced with R175H mutp53 expression vectors to increase the level of mutp53 protein to a comparable level as endogenous mutp53 in SK-BR3 cells transduced with control vectors. Data represent three repeats with similar results. Cells were then used for assays in **b** and **c**. **b,** Ectopic expression of R175H mutp53 partially rescued the impaired AIG in soft agar of SK-BR3 cells resulting from FASN KO. **c,** Ectopic R175H mutp53 expression partially rescued the impaired growth of SK-BR3 orthotopic tumors resulting from FASN KO. Left: the image of collected tumors. Scale bar: 10 mm. Data represent mean ± SD (n = 6 independent experiments in **b** and n=10 mice/group in **c**). One-way ANOVA followed by Tukey's test. Source data are provided as a Source Data file.

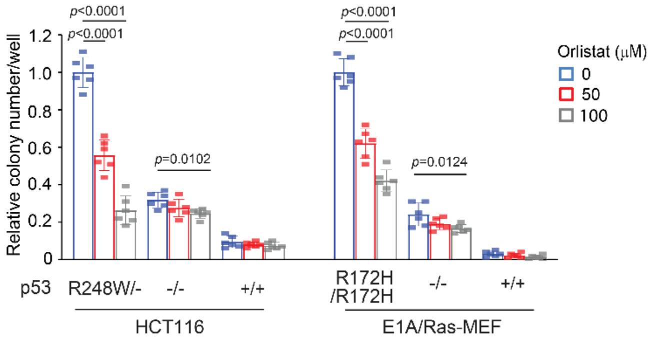

Supplementary Fig. 16. The effect of Orlistat on AIG in HCT116 and MEF cells with different p53 status. Orlistat displayed a more pronounced inhibitory effect on AIG in $p53^{R248W/-}$ HCT116 and E1A/Ras-transformed $p53^{R172H/R172H}$ MEF cells compared with their corresponding $p53^{+/+}$ or $p53^{-/-}$ cells. Cells were treated with or without Orlistat (50 or 100 μM) for 72 h for AIG assays. Data represent mean ± SD (n = 6 independent experiments). One-way ANOVA followed by Dunnett's test. Source data are provided as a Source Data file.

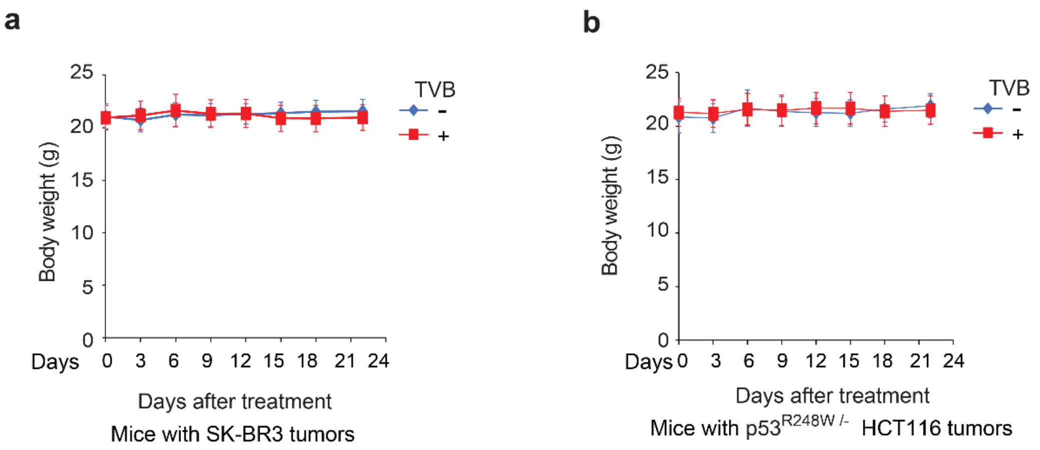

Supplementary Fig. 17. The effect of TVB-3166 treatment on the body weight of tumor-bearing mice. **a**, TVB-3166 treatment did not significantly affect the body weight of female nude mice bearing orthotopic breast tumors formed by SK-BR3 cells. **b**, TVB-3166 treatment did not significantly affect the body weight of nude mice bearing *s.c.* xenograft tumors formed by $p53^{R248W/-}$ HCT116 cells. TVB-3166 was administered once every two days by oral gavage (60 mg/kg of body weight) for 3 weeks. The body weight of the mice was measured and recorded on the days indicated. Data represent mean ± SD. n=10 mice/group. Two-way ANOVA followed by Bonferroni's test. TVB: TVB-3166. Source data are provided as a Source Data file.

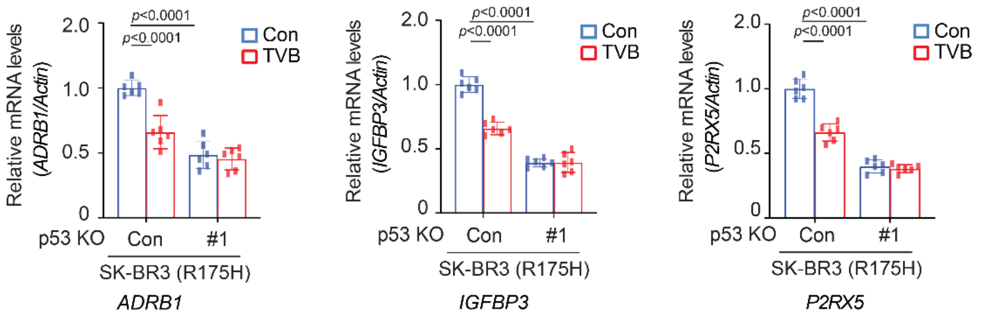

Supplementary Fig. 18. TVB-3166 treatment decreases the expression of mutp53-regulated genes in orthotopic breast tumors formed by SK-BR3 cells in a largely mutp53-dependent manner. Orthotopic breast tumors formed by SK-BR3 cells with or without mutp53 KO were treated with vehicle (Con) or TVB-3166 as described in Fig. 6d and e. The mRNA was extracted from tumors and the expression of known mutp53-regulated genes, including *ADRB1*, *IGFBP3*, and *P2RX5*, was measured by Taqman real-time PCR assays and normalized with Actin. Data represent mean ± SD (n = 6 independent experiments). One-way ANOVA followed by Dunnett's test. TVB: TVB-3166. Source data are provided as a Source Data file.

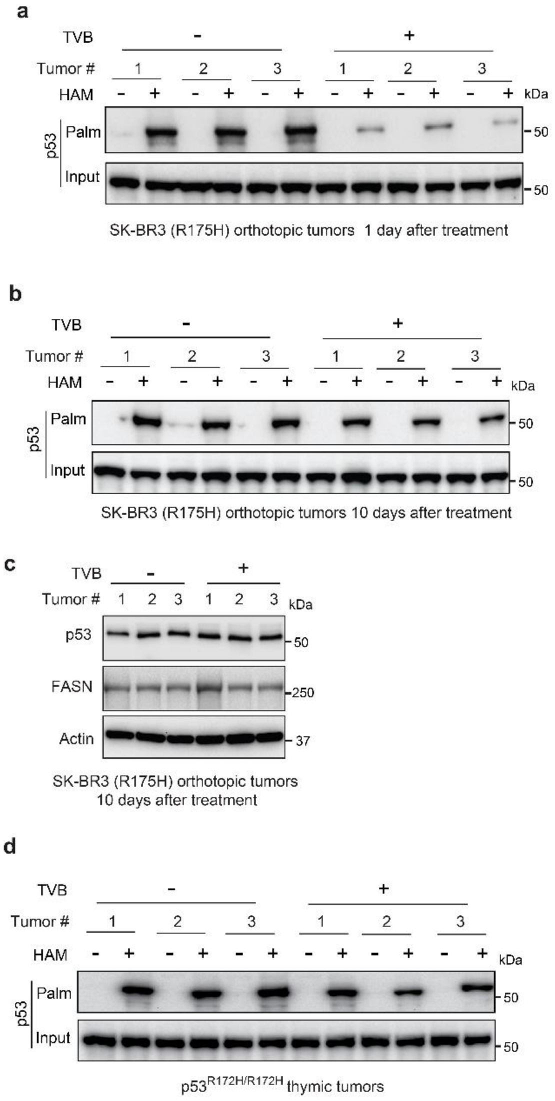

Supplementary Fig. 19. Palmitoylation of mutp53 in tumor tissues of the mice. **a**, The effect of TVB-3166 on palmitoylation of mutp53 in SK-BR3 orthotopic tumors collected one day after the completion of TVB-3166 treatment. The effect of TVB-3166 on mutp53 protein levels in SK-BR3 orthotopic tumors collected one day after the completion of TVB-3166 treatment is presented in Fig 6f. **b**, **c**, The effects of TVB-3166 on palmitoylation (**c**) and protein levels (**d**) of mutp53 in SK-BR3 orthotopic tumors collected 10 days after the completion of TVB-3166 treatment. In **a-c**, mice bearing tumors were treated with or without TVB-3166 for 3 weeks (once every two days by oral gavage; 60 mg/kg of body weight), and tumors were collected for assays at one day (**a**) or ten days after the completion of the treatment (**b**, **c**) to detect mutp53 palmitoylation by ABE assays and mutp53 protein levels by Western blot assays, respectively. **d**. The palmitoylation of mutp53 in thymic tumors of *p53*$^{R172H/R172H}$ mice with or without TVB-3166 treatment was determined by ABE assays. *p53*$^{R172H/R172H}$ mice were treated with or without TVB-3166 for 12 weeks (once every two days by oral gavage; 60 mg/kg of body weight), and tumors were collected after the mice died or reached the humane endpoints, the majority of which occurred far more than 10 days after the completion of TVB-3166 treatment. Tumor samples were analyzed by ABE assays. At least n = 6 tumors/group (in **a-d**) were analyzed and similar results were observed. TVB: TVB-3166. Source data are provided as a Source Data file.

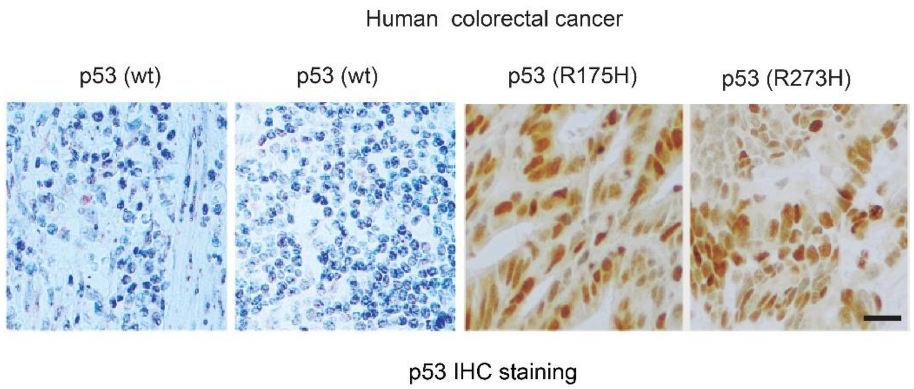

Supplementary Fig. 20. IHC staining of wtp53 and mutp53 in human colorectal cancer specimens. Representative images of wtp53 and mutp53 IHC staining in human colorectal cancer specimens. The p53 mutations in cancer specimens were identified by direct sequencing. Scale bar: 40 μm.
