# Supplementary material for: Lipogenic enzyme FASN promotes mutant p53 accumulation and gain-of-function through palmitoylation
Source: Nat Commun. 2025 Feb 19;16:1762. doi: 10.1038/s41467-025-57099-9 (PMC11839913; doi:10.1038/s41467-025-57099-9)
Supplement: Supplementary file 2 — Description of Additional Supplementary Files [file 41467_2025_57099_MOESM2_ESM.pdf]

Supplementary Data 1

$p53^{-/-}$ HCT116 cells transduced with the control empty vector or vectors expressing wtp53 or R175H mutp53 were employed for analysis. The wtp53 or R175H mutp53 protein was pulled down by anti-p53 (DO-1) beads, and the elutes were subjected to LC-MS/MS analysis. The potential wtp53 or R175H mutp53-interacting proteins are listed with the number of peptides identified by LC-MS/MS analysis. n=1.
