# Supplementary material for: Lipogenic enzyme FASN promotes mutant p53 accumulation and gain-of-function through palmitoylation
Source: Nat Commun. 2025 Feb 19;16:1762. doi: 10.1038/s41467-025-57099-9 (PMC11839913; doi:10.1038/s41467-025-57099-9)
Supplement: Supplementary file 4 — Reporting Summary [file 41467_2025_57099_MOESM4_ESM.pdf]

nature portfolio

Corresponding author(s): Zhaohui Feng

Last updated by author(s): Dec 10, 2024

Reporting Summary

Nature Portfolio wishes to improve the reproducibility of the work that we publish. This form provides structure for consistency and transparency in reporting. For further information on Nature Portfolio policies, see our Editorial Policies and the Editorial Policy Checklist.

Statistics

For all statistical analyses, confirm that the following items are present in the figure legend, table legend, main text, or Methods section.

n/a	Confirmed	
☐	☒	The exact sample size (*n*) for each experimental group/condition, given as a discrete number and unit of measurement
☐	☒	A statement on whether measurements were taken from distinct samples or whether the same sample was measured repeatedly
☐	☒	The statistical test(s) used AND whether they are one- or two-sided<br>*Only common tests should be described solely by name; describe more complex techniques in the Methods section.*
☒	☐	A description of all covariates tested
☐	☒	A description of any assumptions or corrections, such as tests of normality and adjustment for multiple comparisons
☐	☒	A full description of the statistical parameters including central tendency (e.g. means) or other basic estimates (e.g. regression coefficient) AND variation (e.g. standard deviation) or associated estimates of uncertainty (e.g. confidence intervals)
☐	☒	For null hypothesis testing, the test statistic (e.g. *F*, *t*, *r*) with confidence intervals, effect sizes, degrees of freedom and *P* value noted<br>*Give P values as exact values whenever suitable.*
☒	☐	For Bayesian analysis, information on the choice of priors and Markov chain Monte Carlo settings
☒	☐	For hierarchical and complex designs, identification of the appropriate level for tests and full reporting of outcomes
☒	☐	Estimates of effect sizes (e.g. Cohen's *d*, Pearson's *r*), indicating how they were calculated

Our web collection on statistics for biologists contains articles on many of the points above.

Software and code

Policy information about availability of computer code

Data collection	1. qPCR analysis was carried out using the ABI StepOne Plus 96-well Real-Time PCR machine. 2. Western blot analysis was carried out using The ChemiDoc Touch Imaging System (Bio-Rad). 3. Tissue histology images were obtained with an OLYMPUS BX43 microscope. 4. Fluorescent histological images were obtained with a Nikon Confocal microscope using the NIS-Elements -Microscope Imaging Software.
Data analysis	1. Prism 9.0 software (Graphpad) http://www.graphpad.com/scientific-software/ N/A prism/. 2. Western blotting protein bands were quantified with the Image J 1.44 software (NIH).

For manuscripts utilizing custom algorithms or software that are central to the research but not yet described in published literature, software must be made available to editors and reviewers. We strongly encourage code deposition in a community repository (e.g. GitHub). See the Nature Portfolio guidelines for submitting code & software for further information.

Data

Policy information about availability of data

All manuscripts must include a data availability statement. This statement should provide the following information, where applicable:

- Accession codes, unique identifiers, or web links for publicly available datasets
- A description of any restrictions on data availability
- For clinical datasets or third party data, please ensure that the statement adheres to our policy

All data supporting the present study are available within the article and supplementary information files. Source data are provided with this paper.

Research involving human participants, their data, or biological material

Policy information about studies with human participants or human data. See also policy information about sex, gender (identity/presentation), and sexual orientation and race, ethnicity and racism.

Reporting on sex and gender	N/A
Reporting on race, ethnicity, or other socially relevant groupings	N/A
Population characteristics	N/A
Recruitment	N/A
Ethics oversight	N/A

Note that full information on the approval of the study protocol must also be provided in the manuscript.

Field-specific reporting

Please select the one below that is the best fit for your research. If you are not sure, read the appropriate sections before making your selection.

[x] Life sciences [ ] Behavioural & social sciences [ ] Ecological, evolutionary & environmental sciences

For a reference copy of the document with all sections, see nature.com/documents/nr-reporting-summary-flat.pdf

Life sciences study design

All studies must disclose on these points even when the disclosure is negative.

Sample size	Sample size were chosen according to our previous experience and pilot experiments. Sample sizes included at least 3 biological replicates where statistical evaluation was performed. For animal experiments, on the basis of pilot experiments, sample sizes were estimated to provide sufficient numbers of mice in each group for statistical analysis.
Data exclusions	We did not exclude any samples.
Replication	Experiments were repeated as detailed in the figure legends. All attempts at replication were successful. In some cases, multiple cell lines, additional shRNA sequences and two different knockout lines were used to verify the reproducibility of the findings.
Randomization	For in vitro experiments, cells were randomly allocated into control and experimental groups. For in vivo experiments, age and sex-matched mice were randomized into control and experimental groups prior to tumor size measurement and inhibitor treatments. For microscopy, the fields of images were randomly selected.
Blinding	Blinding was not used for animal works because the investigators needed to know the treatment groups in order to perform inhibitor treatments. Blinding was not applicable to the rest of other in vitro experiments (e.g. Western blotting) because the same investigator was doing group allocation during data collection and/or analysis. All IHC-stained slides were examined and scored independently by two investigators who were blinded to the patients' clinical data to avoid subjective biases.

Reporting for specific materials, systems and methods

We require information from authors about some types of materials, experimental systems and methods used in many studies. Here, indicate whether each material, system or method listed is relevant to your study. If you are not sure if a list item applies to your research, read the appropriate section before selecting a response.

Materials & experimental systems

n/a	Involved in the study	
[ ]	[x]	Antibodies
[ ]	[x]	Eukaryotic cell lines
[x]	[ ]	Palaeontology and archaeology
[ ]	[x]	Animals and other organisms
[x]	[ ]	Clinical data
[x]	[ ]	Dual use research of concern
[x]	[ ]	Plants

Methods

n/a	Involved in the study	
[x]	[ ]	ChIP-seq
[x]	[ ]	Flow cytometry
[x]	[ ]	MRI-based neuroimaging

Antibodies

Antibodies used	The following antibodies were used for Western blot:<br>Flag-M2 (F1804, Sigma-Aldrich; 1:20,000 dilution)<br>β-Actin (A5441, Sigma-Aldrich; 1:10,000 dilution)<br>HA (3F10, Roche; 1:1000 dilution)<br>His (sc803, Santa Cruz Biotechnology; 1:1000 dilution)<br>GST (sc-138, Santa Cruz Biotechnology, 1:5000 dilution)<br>FASN (sc55580, Santa Cruz Biotechnology; 1:3000 dilution)<br>Lamin A/C antibody (sc-7292, Santa Cruz Biotechnology; 1:5000 dilution)<br>GAPDH antibody (sc-47724, Santa Cruz Biotechnology; 1:5000 dilution)<br>p53 (CM5, Leica Biosystems; 1:1000 dilution)<br>p53 (sc126, Santa Cruz Biotechnology; 1:2000 dilution)<br>goat anti-mouse (31430, Thermo Fisher Scientific; 1:5,000 dilution)<br>goat anti-rabbit (31460, Thermo Fisher Scientific, 1:5,000 dilution)<br><br>The following antibodies were used for Immunofluorescence:<br>FASN (10624-2-AP, Proteintech, 1;100 dilution)<br>p53 (sc126, Santa Cruz Biotechnology, 1:100 dilution)<br>Flag (F7425, Sigma-Aldrich, 1:500 dilution)<br>The following antibodies were used for IHC:<br>p53 (sc126, Santa Cruz Biotechnology, 1:100 dilution)<br>FASN (sc55580, Santa Cruz Biotechnology; 1:100 dilution)<br>Ki-67 (ab16667, Abcam, 1:200 dilution)
Validation	Validation for commercially available antibodies can be found using the links below:<br>Flag-M2: https://www.sigmaaldrich.com/US/en/product/sigma/f1804<br>β-Actin : https://www.sigmaaldrich.com/US/en/product/sigma/a5441?context=product<br>HA: https://www.sigmaaldrich.com/US/en/product/roche/roahaha<br>His: https://www.scbt.com/p/his-probe-antibody-h-15<br>GST: https://www.scbt.com/p/gst-antibody-b-14<br>FASN: https://www.scbt.com/p/fatty-acid-synthase-antibody-a-5<br>Lamin A/C antibody: https://www.scbt.com/p/lamin-a-c-antibody-636<br>GAPDH: https://www.scbt.com/p/gapdh-antibody-0411<br>p53: https://shop.leicabiosystems.com/us/ihc-ish/ihc-primary-antibodies/pid-p53-protein-cm5 p53: https://www.scbt.com/p/p53-antibody-do-1<br>p53: https://shop.leicabiosystems.com/us/ihc-ish/ihc-primary-antibodies/pid-p53-protein-cm5<br>Ki-67 (ab16667, Abcam): https://www.abcam.com/ki67-antibody-sp6-ab16667.html<br>goat anti-mouse (31430, Thermo Fisher Scientific): https://www.thermofisher.com/antibody/product/Goat-anti-Rabbit-IgG-H-L-Secondary-Antibody-Polyclonal/31430<br>goat anti-rabbit (31460, Thermo Fisher Scientific):https://www.thermofisher.com/antibody/product/Goat-anti-Rabbit-IgG-H-L-Secondary-Antibody-Polyclonal/31460<br>Additionally, all antibodies were validated by using cells with ectopic expression of target genes and/or knockdown of target genes.

Eukaryotic cell lines

Policy information about cell lines and Sex and Gender in Research

Cell line source(s)	SK-BR3, MDA-MB468, and LS1034 cell lines were obtained from American Type Culture Collection (ATCC). The isogenic p53+/+, p53-/- and p53 R248W/- HCT116 cell lines were generous gifts from Dr. Bert Vogelstein (Johns Hopkins University). p53+/+, p53-/- and p53 R172H/R172H MEFs were isolated from 13.5 day embryos of p53+/+, p53-/- and p53 R172H/R172H mice, respectively, according to the standard procedures.
Authentication	Cell were authenticated by short tandem repeat profiling.
Mycoplasma contamination	Cells were regularly tested for mycoplasma using Lookout Mycoplasma PCR detection kit (MP0035, Sigma-Aldrich) and only used when negative.
Commonly misidentified lines<br>(See ICLAC register)	No commonly misidentified cell lines were used.

Animals and other research organisms

Policy information about studies involving animals; ARRIVE guidelines recommended for reporting animal research, and Sex and Gender in Research

Laboratory animals	Mice were maintained at an ambient temperature of 22±1°C and relative humidity 40–60% under a 12h:12h light: dark cycle. For

	orthotopic breast tumor models, 8-week-old female BALB/c athymic nude mice were used. For s.c. xenograft tumor models, 8-week-old BALB/c athymic nude mice (half male and half female) were used. p53 R172H/R172H mice (a gift from Dr. Guillermina Lozano, MD Anderson Cancer Center), p53+/+, and p53-/- mice (The Jackson Laboratory) were used in this study. All mice were scheduled for euthanasia once tumor volume had reached 1,700 mm^3, as indicated in the IACUC protocols. The maximal tumor size of all mice used in this study did not exceed 1,700 mm^3.
Wild animals	No wild animals.
Reporting on sex	Due to the sex-tendency of breast cancer, only female mice were used for orthotopic breast tumor models. Both male and female mice were used for other mouse experiments.
Field-collected samples	No samples collected from the field.
Ethics oversight	Experiments with mice were conducted under the approval of the Institutional Animal Care and Use Committee at Rutgers University (protocol number: 201702554).

Note that full information on the approval of the study protocol must also be provided in the manuscript.

Plants

Seed stocks	N/A
Novel plant genotypes	N/A
Authentication	N/A
